# Psychosocial Work Stress, Resilience and the Risk of Tinnitus—Results from a Population-Based Cohort Study

**DOI:** 10.3390/medicina61122079

**Published:** 2025-11-21

**Authors:** Berit Hackenberg, Julia Döge, Karoline O’Brien, Matthias Nübling, Pavel Dietz, Manfred E. Beutel, Anna Celine Reinwarth, Karl J. Lackner, Oliver Tüscher, Jörn M. Schattenberg, Lukas Hobohm, Thomas Münzel, Philipp S. Wild, Alexander K. Schuster, Irene Schmidtmann, Julian Chalabi, Christoph Matthias, Katharina Bahr-Hamm

**Affiliations:** 1 Department of Otorhinolaryngology, University Medical Center Mainz, 55131 Mainz, Germany; 2 FFAW Freiburg Research Centre for Occupational Sciences, Bertoldstr. 63, 79098 Freiburg, Germany; 3 Institute of Occupational, Social and Environmental Medicine, University Medical Center Mainz, 55131 Mainz, Germany; 4 Department of Psychosomatic Medicine and Psychotherapy, University Medical Center Mainz, 55131 Mainz, Germany; 5 Institute for Clinical Chemistry and Laboratory Medicine, University Medical Center Mainz, 55131 Mainz, Germany; 6 Department of Psychiatry and Psychotherapy, University Medical Center Mainz, 55131 Mainz, Germany; 7 Metabolic Liver Research Center and Medicine, University Medical Center Mainz, 55131 Mainz, Germany; 8 Department of Cardiology—Cardiology I, University Medical Center Mainz, 55131 Mainz, Germany; 9 Preventive Cardiology and Preventive Medicine, Department of Cardiology, University Medical Center Mainz, 55131 Mainz, Germany; 10 German Center for Cardiovascular Research (DZHK), Partner Site Rhine-Mine, 10785 Mainz, Germany; 11 Clinical Epidemiology and Systems Medicine, Center for Thrombosis and Hemostasis, University Medical Center Mainz, 55131 Mainz, Germany; 12 Systems Medicine, Institute of Molecular Biology (IMB), 55128 Mainz, Germany; 13 Department of Ophthalmology, University Medical Center Mainz, 55131 Mainz, Germany; 14 Institute for Medical Biometry, Epidemiology and Informatics, 55131 Mainz, Germany

**Keywords:** tinnitus, stress, work-related, occupational stress, cohort studies

## Abstract

*Background and Objectives*: Tinnitus is a common symptom in otolaryngologic practice. Although its pathophysiology is multifactorial and remains mostly unclear, it can be correlated to stress and psychological comorbidities. The aim of this study was to assess the correlation between the occurrence of tinnitus and psychosocial work stress (measured using the German COPSOQ-III, a validated instrument) in a large working population. *Materials and Methods*: The Gutenberg Health Study is a single-center, prospective, observational cohort study. Participants of working age were included and surveyed using the German COPSOQ-III; they were interviewed regarding the occurrence of tinnitus (yes/no) and stratified according to their resilience (measured using the Brief Resilient Coping Scale). *Results*: A total of 4933 participants of working age were included in the study cohort, in which tinnitus was reported with a prevalence of 26.3%. Participants with tinnitus answered more negatively in all COPSOQ scales, although not all differences were statistically significant. The scales Emotional Demands, Work Privacy Conflicts, Work Environment/Physical Demands and Insecurity over Working Conditions showed especially high differences in means. In addition, all effect scales showed significant differences between participants with and without tinnitus. The prevalence of tinnitus decreased with increasing resilience. *Conclusions*: Tinnitus is a symptom highly correlated with psychosocial work stress. As such, it represents a significant health burden within the working community.

## 1. Introduction

Tinnitus is defined as a sound that is perceived without an external source [1]. It might be experienced as ringing, buzzing or other noises and can occur in one ear, both ears or the head in general; some might even locate it to an external point of origin [1]. Although it is a commonly reported symptom, with a prevalence ranging from 4.1% to 37.2% [2], the etiology of tinnitus is multifactorial and not yet fully understood [3]. Numerous risk factors for tinnitus are known: they can be found within the auditory system, and may be related to other diseases, environmental factors (e.g., occupational and recreational noise exposure) or psychological dysfunctions. Important risk factors within the auditory system are otosclerosis, Ménière’s disease and vestibular schwannoma [1]. Other risk factors include neurological diseases (e.g., meningitis), cardiovascular diseases (e.g., hypertension), metabolic influences (e.g., diabetes mellitus) or structural pathologies (e.g., dysfunction of the temporomandibular joint) [4,5]. Additionally, obesity, smoking, alcohol consumption, previous head injuries and history of arthritis have been suggested in the literature as concomitant risk factors [1]. Even the intake of drugs such as salicylates, quinines, aminoglycoside antibiotics and antineoplastic agents (especially platinum-based ones) can trigger tinnitus [6]. While genetic predisposition to tinnitus has been discussed in the literature [7], Lopez-Escamez et al. found only one genotyping study reporting an association between a candidate gene (KCNE1) and tinnitus in their review [8,9]. Hearing impairment remains the most prevalent condition associated with tinnitus [10], with a retrospective study by Zhang et al. demonstrating that 77.8% of tinnitus patients also presented hearing loss in pure tone audiometry [11]. Similarly, Mazurek et al. reported an 83% prevalence of high-frequency hearing loss in a population of 531 tinnitus patients [12].

Due to the correlation between hearing impairment and tinnitus, it is assumed that a cochlear deficit is the origin of tinnitus. Studies showing that patients with profound hearing loss can experience a significant improvement in their tinnitus if they are fitted with hearing aids or receive a cochlear implant support this assumption [13,14]. Other central non-auditory processes presumably play a major role in the persistence of tinnitus [15]. Tinnitus may result from hyperactive neuronal activity in the central auditory region, and thus, might be caused by maladaptive neuroplasticity [16,17]. It is assumed that the negative experiences associated with the sensation of tinnitus also stimulate the limbic system [18].

Stress and negative emotions play an important role in this complex processing. It is recognized that stress is closely related to the occurrence of tinnitus, although it remains unclear whether stress is a risk factor or a consequence of tinnitus [19,20]. Various models have been developed in an attempt to explain the interactions between stress and tinnitus [21]. Mazurek et al. proposed to distinguish between a tinnitus sound originating from medical risk factors (see above) and reactive tinnitus-related distress [4], with the latter being closely linked to psychological influences. Pre-existing emotional distress can cause tinnitus-related distress. On the other hand, psychological distress can also be triggered by tinnitus. Stress is thought to stimulate the production and release of cortisol; in turn, high cortisol levels influence the potassium concentration in the cochlea [22]. In addition, cortisol may influence the neuronal plasticity of the auditory system [22]. Transferring this concept to the bedside remains difficult due to the varying definitions of stress. In general, stress is defined as a feeling of emotional and mental strain, which usually occurs in connection with a particularly demanding task or event [19]. Stress therefore occurs due to an individual’s interaction with their environment, and arises from a discrepancy between internal or external strain and an individual’s resources to overcome those [23]. Excessive demands with insufficient resources, negative evaluation of experiences and a lack of recognition for what has been achieved can lead to stress [24]. While stress influences the biophysiological balance by interfering with the hypothalamic–pituitary–adrenal and sympathetic–adrenal–medullary axes [19], perceived stress is the usual outcome measured in clinical studies. In their recent review, Elarbed et al. found that tinnitus patients tend to report louder and/or more bothersome tinnitus when they feel stressed [19].

Resilience is a process of adapting to trauma or other life-changing events or conditions. It is based on family support, personal characteristics (i.e., genetic predispositions) and external support systems [25], and should be understood as a dynamic system. Resilience has been found to serve as a protective factor against anxiety and depression, and has a positive influence on sleep quality [26,27]. Studies on the relationships between tinnitus and resilience are scarce. Among 61 patients with tinnitus, Xin et al. observed lower resilience in comparison with patients from primary health services and the general population [25]. In a study on 4705 individuals, Wallhäuser-Franke et al. found that resilience only had an indirect effect on tinnitus-related distress [28].

High job strain has been shown to be a major contributor to the overall mental stress level [29,30]. In occupational science, a distinction is made between various influencing factors, including work load, stress (as the sum of all external factors) and strain (as the effect that stress has on employees depending on individual factors). Furthermore, strain can lead to disease as a consequence. Research models generally do not assume the relationship between stress and strain to be deterministic, but deem it to be influenced by individual factors (i.e., resilience) [31]. With the recognition of stress as an important risk factor for disease, the possible causes of stress need to be examined more closely.

In this study, we examine the possible influence of work-related stressors on tinnitus as a potential consequence. The aim of this study is to investigate the correlation between psychosocial working conditions, assessed using the Copenhagen Psychosocial Questionnaire (COPSOQ), and the occurrence of tinnitus in a large population-based cohort. Furthermore, as a secondary outcome, we analyzed the influence that resilience can have on this interaction as a protective factor. For this purpose, the Brief Resilient Coping Scale (BRCS) was used, as a scale that measures resilience regarding a person’s ability to cope with stress adaptively [32].

## 2. Materials and Methods

### 2.1. Gutenberg Health Study (GHS)

This cross-sectional study was based on the Gutenberg Health Study (GHS). The GHS involves a large population-based cohort located at the University Hospital Mainz, Germany. It was initiated in 2007 and included 15,000 participants in its core cohort. To participate, residents of Mainz and its district Mainz-Bingen were randomly drawn from the residents’ registration office. All participants were stratified by sex and location (rural vs. urban). After five years, participants were followed-up through a computer-assisted interview. Another five years later, at the 10-year follow-up (10-FU), all participants were invited to re-visit the study site. In addition to the core cohort, new participants in the age groups of 25 to 44 years (young cohort, *n* = 4000) and 75 to 85 years (senior cohort, *n* = 1000) were recruited. Exclusion criteria for participating in the GHS were insufficient knowledge of the German language and disabilities that prevented participants from attending the study site for examination.

Participants completed different interviews and examinations from different fields of medicine. During the interviews, participants were asked about their working conditions. They stated their working hours per week, overtime hours per week and whether they conducted night work (and, if so, they also stated their night work hours per month). In the 10-FU, an otologic survey and audiometric testing were added to the examinations. Participants were asked: “Do you suffer from ringing in the ears (tinnitus)?” (yes/no). Hearing status was tested via pure-tone audiometry for air and bone conduction using the Auritec^®^ AT1000 clinical audiometer in a soundproof booth. Hearing impairment was classified according to the revised WHO classification [33]. Following the WHO grading system for hearing impairment, the hearing threshold for each participant was calculated as the average hearing loss in dB (decibel) across the frequencies 0.5/1/2/4 kHz. The mean value derived from these four frequencies for the better hearing ear was taken, referred to as the mean hearing impairment for each participant in the following. The *p*-values for differences in mean hearing impairment (tinnitus yes vs. no) were calculated using the Wilcoxon test, as the skewness was greater than 1.

In addition, participants were asked to complete the COPSOQ III and BRCS questionnaires. The COPSOQ is an international instrument for research and risk assessment of psychosocial working conditions [34]. With the two requirements of providing internationally comparable data and being sensitive to cultural differences, the COPSOQ comprises internationally mandatory core questions and additional questions with national relevance [34]. In 2021, a German version of the third edition—the COPSOQ III—was published [35]. In particular, the COPSOQ III is a validated questionnaire measuring psychosocial working conditions [36]; based on different theoretical models, it is an instrument covering a broad variety of working conditions [37]. The German version of the COPSOQ III includes five domains: Demands, Influence and Possibilities for Development, Social Relations and Leadership, Additional Factors, and Effects [35]. Together, these five domains comprise 31 scales and 84 single items. Depending of the content of each scale, higher scores can either be favorable or unfavorable. In line with the previous literature, the COPSOQ values were scaled to range from 0 (minimum) to 100 (maximum). Each scale’s score is calculated as the mean of all items of that scale [38].

The BRCS contains four items and is a unidimensional outcome measure designed to assess an individual’s level of resilience (i.e., the extent to which they can cope with stress). The items are rated on a five-level scale, with answers ranging from 1 = “Does not describe me at all” to 5 = “Describes me very well.” Participants can be categorized, based on their sum score, as having either low, medium or high resilience [32].

### 2.2. Study Design

The study design was cross-sectional. Participant charts were retrospectively reviewed. Participants were excluded from this study if data on tinnitus or COPSOQ and BRCS questionnaire scores were missing. Furthermore, participants aged above 65 years were excluded to examine only those that are currently working.

### 2.3. Descriptive Analysis

The prevalence of tinnitus (y/n) and the participants’ workload (working hours, overtime hours, night work) was described for women and men. In addition, hearing thresholds according to the WHO grading system (see above) were described for different age groups and depending on the presence of tinnitus (y/n). Means in all COPSOQ scales were calculated for participants with and without tinnitus. Continuous variables are described as median (Q1, Q2) and were tested using a U-test. Discrete variables are described as relative and absolute frequencies and were tested with a chi-squared test.

### 2.4. Correlation Analysis

A stepwise selection regression model was used to select the most statistically significant predictors from the COPSOQ. In a second step, an elastic net regression model was used to select a combination of predictors for the logistic regression. A logistic regression model was then constructed to measure the influence of COPSOQ scales on tinnitus occurrence in relation to sex, age and resilience.

The study was approved by the local institutional review board (Ethics Commission of the State Chamber of Physicians of Rhineland-Palatine, reference no. 837.020.07) and was conducted in full compliance with the Declaration of Helsinki. Written informed consent was obtained from all subjects before participation in the study.

## 3. Results

**Figure 1:**
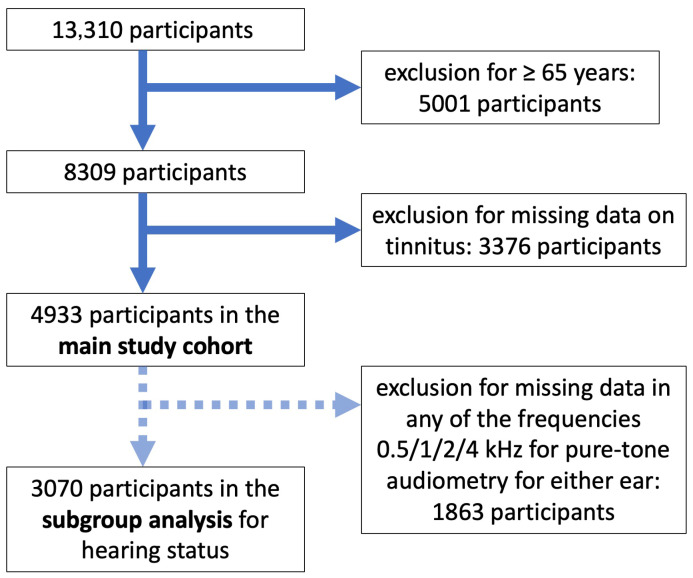
Flowchart for participant selection.

Of the 13,310 participants returning to the 10-FU or newly recruited, 5001 were excluded due to being aged 65 or above. Of the remaining 8309 participants, 3376 were further excluded due to missing information on tinnitus. Hence, 4933 participants of working age were included in the main study cohort. In a subgroup analysis, hearing loss was evaluated according to age, sex and tinnitus (y/n). A further 1863 participants were excluded due to missing data in any of the frequencies (0.5/1/2/4 kHz) in pure-tone audiometry. Figure 1 details the participant selection process. Missing values for single items in the COPSOQ and BRCS questionnaires did not lead to study exclusion. See Figure S1 in the Supplementary Materials for information on missing data in COPSOQ and BRCS.

### 3.1. Descriptive Analysis

Of the 4933 participants, 51.1% were women (48.9% men, *p* = 0.47). The median age was 53 years (Q1: 46 years/Q3: 59 years). Tinnitus was reported with a prevalence of 26.3%, with a significantly higher prevalence in men (29.4% in men, 23.3% in women; *p* < 0.0001). Median working hours per week was 38.5 h/week (Q1: 30 h/week, Q3: 40 h/week), with men working significantly more hours per week (men: 40 h/week, women: 32 h/week, *p* < 0.0001). In the study cohort, the median overtime hours per week was reported as 2 h (Q1: 0 h, Q3: 5 h), with no significant sex differences in reported overtime hours per week. Of all participants, 11.5% indicated conducting night work, with men significantly more often reporting to do so (men: 15.6%, women: 7.4%, *p* < 0.0001). The descriptive statistics are summarized in Table 1.

The average hearing impairment was calculated, according to the WHO classification, as average hearing loss in dB across the frequencies 0.5/1/2/4 kHz. Mean hearing impairment increased with age. In females aged 25 to 34, hearing impairment was significantly greater in those who did not report tinnitus than in participants who reported tinnitus (mean hearing impairment: tinnitus yes, 7.16 dB; tinnitus no, 7.48 dB; *p* = 0.029). At age 45 or older, hearing impairment was greater among participants with tinnitus, although this difference was only significant among women (mean hearing impairment age 45–54: tinnitus yes, 14.6 dB; tinnitus no, 11.2 dB; *p* < 0.0001; mean hearing impairment age 55–64: tinnitus yes, 17.2 dB; tinnitus no, 15.9 dB; *p* = 0.006) (Table 2).

### 3.2. Comparative Analysis

#### 3.2.1. Results of the COPSOQ (Depending on Tinnitus Yes/No)

In the Demands domain of the COPSOQ, participants with tinnitus presented significantly higher mean scores in the scales Emotional Demands, Hiding Emotions and Work Privacy Conflicts. In the domain Influence and Possibilities for Development, only the scale Degrees of Freedom (Breaks/Holidays) showed a significant difference between participants with and without tinnitus, with participants having tinnitus reporting a lower mean score. In the domain Social Relations and Leadership, participants with tinnitus reported significantly lower mean scores in the scales Predictability of Work, Support at Work, Quantity of Social Relations, Sense of Community, Trust and Justice, and Recognition. In addition, their mean scores in the scales Role Conflicts and Unfair Treatment were significantly higher. All three scales in the domain Additional Factors (Work Environment/Physical Demands, Job Insecurity, Insecurity over Working Conditions) were reported with significantly higher mean scores among participants with tinnitus. Furthermore, all mean scores in the scales summarized in the domain Effects were significantly different between participants with and without tinnitus. The Intention to leave Profession/Job, Burnout Symptoms, Presenteeism and Inability to Relax scales were higher among participants with tinnitus, while Job Satisfaction, General Health and Work Engagement were lower among participants with tinnitus (see Table 3). Table S1 in the Supplementary Materials details single items for all COPSOQ III scales.

The greatest differences in means (Δmeans) between participants with and without tinnitus were found in the outcome scales General Health and Burnout Symptoms, with participants with tinnitus reporting lower means in General Health (Δmeans = 5.72) and higher means in Burnout Symptoms (Δmeans = 5.74). In the predictor scales, the scales Emotional Demands, Work Privacy Conflicts and Job Insecurity presented the biggest differences in means (see Table 3).

#### 3.2.2. Results of the BRCS (Depending on Tinnitus Yes/No)

The BRCS total score, as an average of the scores for the four BRCS items, was significantly lower among participants with tinnitus (see Table 4).

**Figure 2:**
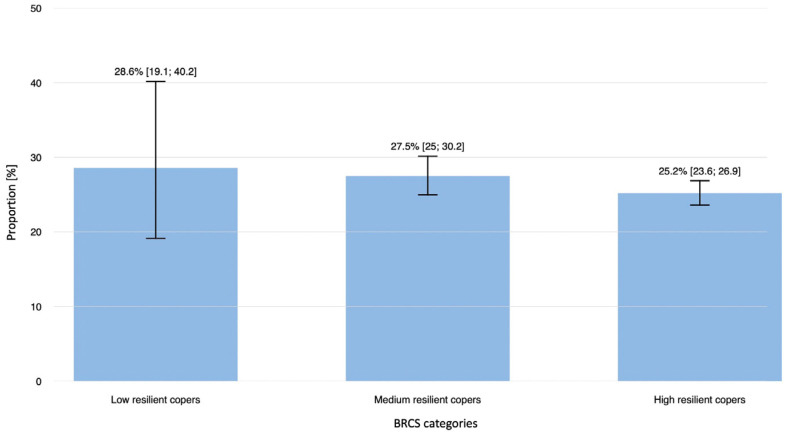
Prevalence of tinnitus depending on resilience category (low, medium and high resilient copers).

Participants with tinnitus reported lower mean scores in all four BRCS items, although this difference was only statistically significant for the statement “I believe that I can grow in positive ways by dealing with difficult situations.” In particular, lower scores indicate lower resilience. Overall, the prevalence of tinnitus was highest among participants with low resilience and decreased gradually in those with low to medium to high resilience (Figure 2). This trend was statistically significant (*p*-value: 0.0052, chi-squared test for trend).

### 3.3. Correlation Analysis

Using the elastic net regression model, the scales Emotional Demands, Work Privacy Conflicts, Feedback, Work Environment/Physical Demands and Insecurity over Working Conditions were chosen for the following logistic regression model.

After adjusting for sex and age, the scales Emotional Demands, Work Privacy Conflicts, Work Environment/Physical Demands, and Insecurity over Working Conditions were statistically associated with the presence of tinnitus (Table 5).

Individuals with medium resilience had a 20.3% increased risk of suffering from tinnitus than those with high resilience (odds ratio 1.203 [1.056;1.371], *p*-value = 0.0055). The effect of low resilience status was statistically not significant, due to the small sample size (Table 6).

When including the top COPSOQ predictor scales as chosen by the elastic net regression model, there was no statistical association between resilience and tinnitus (Table 7).

The effects of the COPSOQ scales Work Privacy Conflicts, Work Environment/Physical Demands, and Insecurity over Working Conditions remained statistically significant after adjusting for resilience. However, this was not the case for the scale Emotional Demands. Participants with tinnitus scored 0.6% higher on the scale Work Privacy Conflicts and 0.7% higher on the scales Work Environment/Physical Demands and Insecurity over Working Conditions, when compared with those without tinnitus. Coping status did not influence this correlation (Table 6). When taking working hours/week, night work (yes), overtime hours/week and hearing impairment (20–64.9 dB) into the model, all four did not show a significant effect on tinnitus presence (working hours/week: OR 0.992 [0.969;1.015], *p* = 0.48; night work (yes): OR 1.583 [0.835;3.002], *p* = 0.16; overtime hours/week: OR 1.024 [0.991;1.058], *p* = 0.16; hearing impairment (20–64.9 dB: OR 1.511 [0.794;2.875], *p* = 0.21). Only four participants showed a hearing impairment greater than 64.9 dB (severe to complete hearing impairment). Due to this small sample size, hearing impairment > 64.9 dB was not modeled in the logistic regression model.

## 4. Discussion

With this study, we showed that the presence of tinnitus is correlated with psychosocial work stress as measured by the validated instrument COPSOQ III. Participants with tinnitus reported their work to be more demanding and saw less freedom in their work, as opposed to those without tinnitus. Furthermore, their work seemed less predictable, they reported more role conflicts, less support at work, had fewer social relations at work, a smaller sense of community and scored significantly lower in the perceived fairness, trust and recognition they experienced at their work site. In addition, participants with tinnitus rated their work environment as more demanding and reported higher insecurities in their job and working conditions. This led to significantly lower scores in all effect scales. Participants with tinnitus had a higher intention to leave their job, lower job satisfaction, lower general health, higher burden of burnout symptoms, higher presenteeism, lower ability to relax and lower work engagement.

This is the first study to correlate psychosocial work stress, as assessed by the COPSOQ III instrument, with the occurrence of tinnitus. However, the link between tinnitus and stress in general has already been discussed in the literature. A recent review on the correlation between stress and tinnitus by Elarbed et al. showed that most of the studies have examined stress in tinnitus populations [19,39,40,41,42,43,44]. In these studies, the number of participants ranged from 72 to 311 [39,40]. All studies found a positive correlation between perceived stress and tinnitus. Only in one study was perceived stress also measured with the PSQ-20 (Perceived Stress Questionnaire 20; German version) in a healthy control group (*n* = 19) [45], with participants who suffered from tinnitus reporting a higher level of perceived stress than the healthy controls. The measurement of stress varied between studies. Most studies assessed perceived stress using standardized instruments such as the PSQ, Perceived Stress Scale (PSS) or Depression Anxiety Stress Scale (DASS) [19], while stress was measured using stimulus-based methods or biophysiological stress markers in fewer studies [19].

A report from the Swedish Work Environment Survey by Hasson et al. included self-reports from 9756 working individuals [46], indicating a significant association between hearing problems and work-related stress factors as well as health outcomes. In their report, they combined the occurrence of self-reported hearing complaints and/or tinnitus into one variable and differentiated the results not only for tinnitus. However, they reported poorer self-rated health and higher burnout symptoms associated with hearing problems, which is consistent with the results of our study.

Abbott et al. reported on the effectiveness of therapist-supported internet-based cognitive behavioral therapy training for tinnitus distress in a working community in an Australian industrial setting [47]. As part of this study, among other outcomes, they assessed occupational stress as measured by the Occupational Stress Inventory—Revised (OSI-R) [48], which is divided into three scales: Occupational Role Questionnaire (ORQ), Personal Strain Questionnaire (PSQ) and the Personal Resources Questionnaire (PRQ). T-scores of 70 or more are interpreted as a high probability of maladaptive stress or strain. The mean values in all three scales were between 46.14 and 52.39 before the study and did not indicate an increased level of occupational stress in the tinnitus participants. Inclusion criteria were having had tinnitus for at least three months and having been diagnosed with tinnitus by a health professional, although most participants had not sought treatment for their tinnitus prior to the study. As there were no healthy controls, it remains unclear whether occupational stress was elevated in comparison with workers without tinnitus.

In this study, we found the largest mean differences (Δmeans) in predictor scales in the scales Emotional Demands, Work Privacy Conflicts and Job Insecurity, with participants with tinnitus responding more negatively. The scales Work Privacy Conflicts and Job Insecurity have been shown to be highly correlated with stress symptoms [49]. The scale Job Insecurity addresses the concern of losing one’s job. Tinnitus could be correlated with these fears, as it is also known to be associated with anxiety [50]. Furthermore, the occurrence of tinnitus was significantly correlated with poorer health outcomes in General Health and Burnout Symptoms. This is in line with previous studies reporting burnout to be more prevalent in individuals with especially bothersome tinnitus [46,51].

The limitations of this study include the fact that tinnitus was assessed as a yes/no question and was not captured using a standardized questionnaire (e.g., the Tinnitus Handicap Inventory). The COPSOQ is designed to capture outcomes that predict psychosocial working conditions [36]. Therefore, we reported on the correlation between the two outcomes. With the study having a cross-sectional design, all results were evaluated at a single point of time. Hence, this study describes significant correlations, but lacks causality. Further longitudinal studies will be needed to explore possible causal effects. In addition, information on the job/occupation of each participant was lacking, which means that the findings cannot be related to separate fields of employment.

However, the strength of this study is its large cohort size and the population-based design. As all previously described studies observed stress levels within a pre-selected tinnitus population or lacked differentiation between tinnitus and hearing impairment, our study is the first to investigate the association between occupational stress and tinnitus occurrence in a population-based setting. As such, the selection bias associated with participants seeking clinical treatment for their tinnitus was significantly decreased. In addition, a well-established instrument for measuring work-related stress was used in this study, as the COPSOQ is a widely used questionnaire that emphasizes the multidimensional aspects of psychosocial work stress. As such, it has been applied in many different settings [34].

Our study showed that tinnitus is closely associated with work-related stress. Although not all differences were statistically significant, participants with tinnitus answered more negatively in each scale. Resilience, on the other hand, was inversely correlated with the prevalence of tinnitus and might influence this relationship as a protective factor.

## 5. Conclusions

Tinnitus represents an important burden within the working population. Longitudinal, population-based studies are needed to shed more light on the causality of this correlation to limit stress and decrease somatic stress reactions such as tinnitus.

## Figures and Tables

**Table 1:** Descriptive statistics of the study cohort.

	All (*n* = 4933)	Men (*n* = 2412)	Women (*n* = 2521)	*p*-Value
**Age (years), median (Q1/Q3)**	53.0 (46.0/59.0)	53.0 (47.0/59.0)	53.0 (46.0/59.0)	0.47
**Tinnitus yes, prevalence (%)**	26.3%	29.4%	23.3%	<0.0001 *
**Working hours/week, median (Q1/Q3)**	38.5 (30.0/40.0)	40.0 (38.0/40.0)	32.0 (22.0/40.0)	<0.0001 *
**Overtime hours/week, median (Q1/Q3)**	2.0 (0/5.0)	2.0 (0/5.0)	2.0 (0/5.0)	
**Night work yes, prevalence (%)**	11.5%	15.6%	7.4%	<0.0001 *
**Night work hours/month, median (Q1/Q3)**	4.0 (2.0/8.0)	5.0 (3.0/8.08)	3.0 (1.0/6.0)	0.031 *

* represents *p*-values with statistical significance.

**Table 2:** Mean hearing loss by sex, age group and tinnitus (yes/no).

Age Group in Years	Tinnitus (Yes/No)	Male		Female	
		Mean Hearing Loss in dB * (SD **)	n (Missing)	Mean Hearing Loss in dB * (SD **)	n (Missing)
**25–34**	Yes	7.48 (3.89)	15 (12)	7.16 (3.16)	22 (23)
	No	7.09 (5.14)	88 (72)	7.48 (3.92)	87 (59)
	*p*-value	0.84		0.029 ***	
**35–44**	Yes	8.66 (4.97)	40 (35)	10.8 (7.58)	36 (27)
	No	9.24 (6.65)	159 (97)	8.06 (4.28)	182 (132)
	*p*-value	0.66		0.19	
**45–54**	Yes	14.5 (8.78)	136 (100)	14.6 (10.2)	117 (87)
	No	11.7 (6.22)	371 (206)	11.2 (5.39)	448 (244)
	*p*-value	0.94		<0.0001 ***	
**55–64**	Yes	21.6 (36.0)	220 (150)	17.2 (9.38)	144 (132)
	No	16.3 (8.74)	476 (235)	15.9 (8.45)	529 (252)
	*p*-value	0.72		0.0060 ***	

* means hearing impairment across the frequencies 0.5/1/2/4 kHz in dB calculated for the better hearing ear. ** SD—standard deviation, *** values represent significance with *p* < 0.05.

**Table 3:** Means of COPSOQ III scores by scale and tinnitus (yes/no). Scores range from 0 = minimum to 100 = maximum.

	Scale	Participants with Tinnitus—Mean Score	Participants Without Tinnitus—Mean Score	ΔMeans	*p*-Value	*n*
**Demands**	Quantitative Demands	46.17	45.25	0.92	0.12	3989
	Emotional Demands	42.22	38.89	3.33	0.0012 *	4073
	Hiding Emotions	36.97	34.39	2.58	0.010 *	4075
	Work Privacy Conflicts	29.22	25.24	3.98	<0.0001 *	4077
	Dissolution	33.00	31.30	1.70	0.057	4042
**Influence and Possibilities for Development**	Influence at Work	58.16	59.04	0.88	0.39	4070
	Degrees of Freedom (Breaks/Holidays)	72.37	74.14	1.77	0.016 *	4078
	Possibilities for Development	70.28	71.26	0.98	0.25	4058
	Meaning of Work	75.75	77.15	1.40	0.077	4072
	Commitment to Workplace	62.27	63.61	1.34	0.08	4067
**Social Relations and Leadership**	Predictability of Work	62.05	64.27	2.22	0.0043 *	4040
	Role Clarity	76.87	77.66	0.79	0.19	4035
	Role Conflicts	38.39	36.58	1.81	0.019 *	4032
	Quality of Leadership	55.25	56.39	1.14	0.15	3951
	Support at Work	67.47	69.75	2.28	0.00030 *	3993
	Feedback	49.42	50.72	1.30	0.051	3989
	Quantity of Social Relations	64.78	67.04	2.26	0.021 *	4002
	Sense of Community	76.45	78.05	1.60	0.0031 *	3980
	Unfair Treatment	31.33	29.50	1.83	0.0026 *	4000
	Trust and Justice	64.21	66.01	1.80	0.0051 *	3804
	Recognition	56.49	58.99	2.50	0.012 *	3857
**Additional Factors**	Work Environment/Phys. Demands	39.67	37.12	2.55	<0.0001 *	4040
	Job Insecurity	20.35	17.26	3.09	<0.0001 *	4024
	Insecurity over Working Conditions	17.35	14.42	2.93	<0.0001 *	3988
**Effects**	Intention to leave Profession/Job	17.35	13.46	3.89	<0.0001 *	4017
	Job Satisfaction	68.73	71.23	2.50	<0.0001 *	3865
	General Health	70.59	76.31	5.72	<0.0001 *	4030
	Burnout Symptoms	42.47	36.73	5.74	<0.0001 *	4034
	Presenteeism	35.82	32.37	3.45	0.00013 *	4052
	Inability to Relax	42.60	38.97	3.63	0.00024 *	4049
	Work Engagement	65.37	67.24	1.87	0.0025 *	4058

* represents *p*-values with statistical significance.

**Table 4:** Brief Resilient Coping Scale—items and means by tinnitus (yes/no).

BRCS Item	Mean (Total)	Mean (Tinnitus)	Mean (No Tinnitus)	*p*-Value	*n*
**I look for creative ways to alter difficult situations.**	77.26	76.58	77.50	0.062	4083
**Regardless of what happens to me, I believe I can control my reaction to it.**	69.81	68.61	70.22	0.065	4086
**I believe that I can grow in positive ways by dealing with difficult situations.**	74.46	72.93	74.99	0.0049 *	4090
**I actively look for ways to replace the losses I encounter in life.**	73.38	70.24	74.48	0.78	4060
**BRCS Total score**	71.94	70.82	72.33	0.0033 *	4016

* represents *p*-values with statistical significance.

**Table 5:** Logistic regression model. Odds ratios (OR) to examine the association between the presence of tinnitus (independent variable) and COPSOQ predictor scales (dependent variables), adjusted for sex and age.

Tinnitus	OR [95% CI]	*p*-Value
Emotional Demands	1.003 [1.000;1.005]	0.044 *
Work Privacy Conflicts	1.005 [1.001;1.008]	0.0052 *
Feedback	0.998 [0.995;1.002]	0.32
Work Environment/Physical Demands	1.007 [1.002;1.012]	0.0023 *
Insecurity over Working Conditions	1.008 [1.004;1.012]	<0.0001 *
***n*** = 3885		AUC 0.606

OR—odds ratio, CI—confidence interval, * represents *p*-values with statistical significance.

**Table 6:** Logistic regression model. Odds ratios (OR) to examine the association between the presence of tinnitus (independent variable) and coping status according to the BRCS (dependent variable), adjusted for sex and age.

Tinnitus	OR [95% CI]	*p*-Value
Sex (Women)	0.738 [0.654;0.832]	<0.0001 *
Age	1.021 [1.015;1.027]	<0.0001 *
Low resilience	1.152 [0.734;1.810]	0.54
Medium resilience	1.203 [1.056;1.371]	0.0055 *
***n*** = 4016		AUC 0.582

OR—odds ratio, CI—confidence interval, * represents *p*-values with statistical significance.

**Table 7:** Logistic regression model. Odds ratios (OR) to examine the association between the presence of tinnitus (independent variable), COPSOQ predictor scales (dependent variables) and coping status according to the BRCS (dependent variable), adjusted for sex and age.

Tinnitus	OR [95% CI]	*p*-Value
Sex (Women)	0.695 [0.589;0.821]	<0.0001 *
Age	1.022 [1.013;1.032]	<0.0001 *
Low-Resilience Copers	0.898 [0.467;1.728]	0.75
Medium-Resilience Copers	1.042 [0.870;1.248]	0.65
Emotional Demands	1.002 [0.999;1.005]	0.21
Work Privacy Conflicts	1.006 [1.002;1.010]	0.0025 *
Feedback	0.999 [0.995;1.003]	0.55
Work Environment/Physical Demands	1.007 [1.002;1.012]	0.010 *
Insecurity over Working Conditions	1.007 [1.003;1.011]	0.0019 *
***n*** = 3191		

OR—odds ratio, CI—confidence interval, * represents *p*-values with statistical significance.

## Data Availability

The datasets presented in this article are not readily available because the data are part of a large ongoing population-based cohort study and are part of different study groups` work. Requests to access the datasets should be directed to the corresponding author.

## Supplementary Materials

The following supporting information can be downloaded at: https://www.mdpi.com/article/10.3390/medicina61122079/s1, Figure S1. Missing data on single COPSOQ scales and the BRCS. Table S1. All items of the COPSOQ III.

## Abbreviations

The following abbreviations are used in this manuscript:

BRCS	Brief Resilient Coping Scale
DASS	Depression Anxiety Stress Scale
COPSOQ	Copenhagen Psychosocial Questionnaire
GHS	Gutenberg Health Study
OR	Odds Ratio
ORQ	Occupational Role Questionnaire
OSI-R	Occupational Stress Inventory—Revised
PRQ	Personal Resources Questionnaire
PSQ	Personal Strain Questionnaire
PSS	Perceived Stress Scale
WHO	World Health Organization
