# Supplementary material for: Psychosocial Work Stress, Resilience and the Risk of Tinnitus—Results from a Population-Based Cohort Study
Source: Medicina (Kaunas). 2025 Nov 21;61(12):2079. doi: 10.3390/medicina61122079 (PMC12734541; doi:10.3390/medicina61122079)
Supplement: Supplementary file 1 [file medicina-61-02079-s001.zip › medicina-3955492-supplementary.pdf]

Supplementary Materials

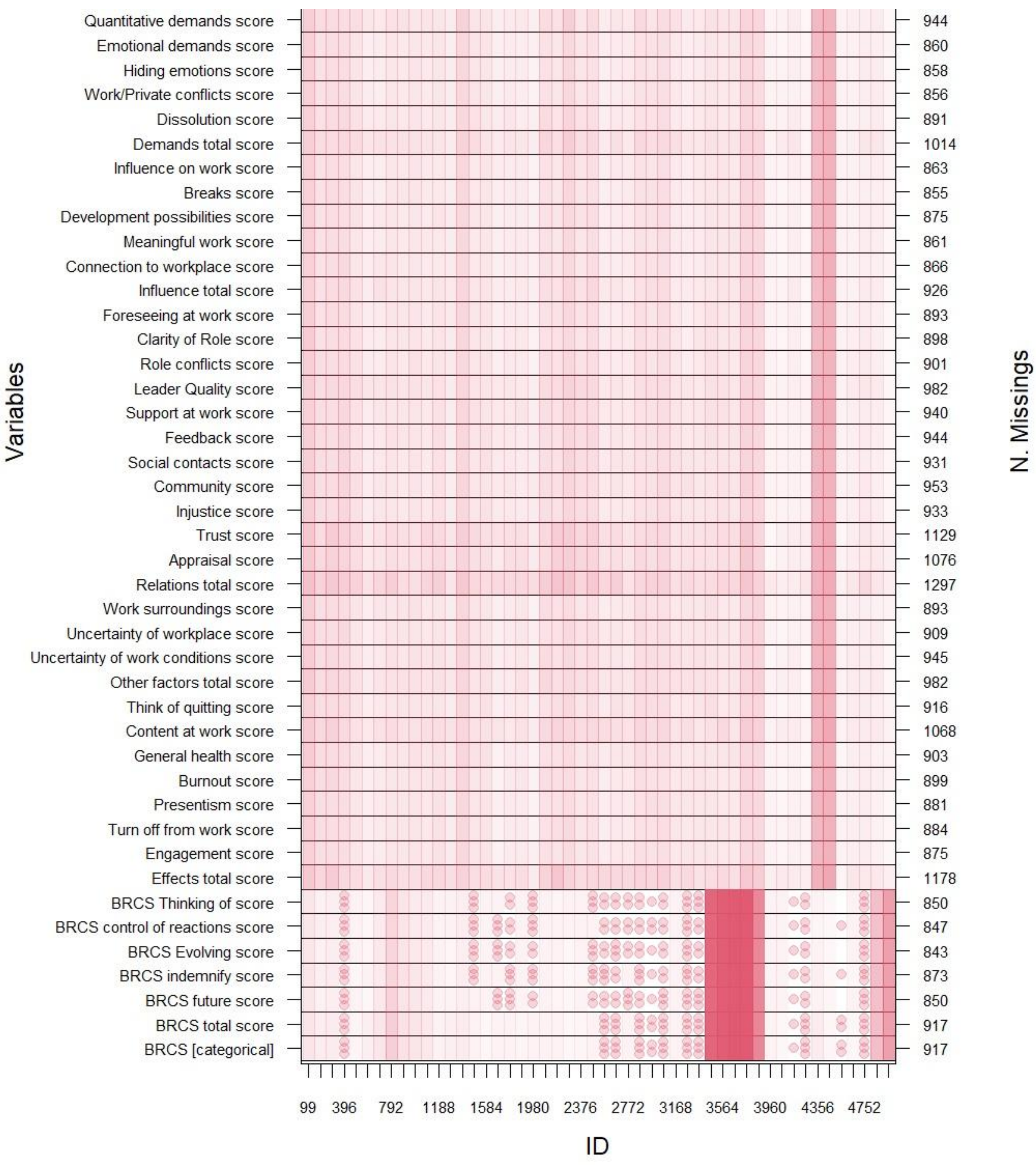

Figure S1. Missing data on single COPSOQ scales and the BRCS.

Table S1. All items of the COPSOQ III

	Scale	Number of items	Items	Response option*
**Demands**	Quantitative Demands	5	Do you have to work very fast?	1
			Do you work at a high pace throughout the day?	1
			How often do you not have time to complete all your work tasks?	1
			Do you get behind with your work?	1
			Do you have to work overhours?	1
	Emotional Demands	2	Do you have to deal with other people´s personal problems as part of your work?	1
			Is your work emotionally demanding?	2
	Hiding Emotions	2	Does your work require that you hide your feelings?	2
			Does your work require that you do not state your opinion?	2
	Work Privacy Conflicts	4	The demands of my job interfere with my personal and family life.	2
			I have to change plans for personal or family activities because of work commitments.	2
			My job takes up so much energy that it has a negative impact on my personal life.	2
			My job takes up so much time that it has a negative impact on my personal life.	2
	Dissolution	2	I also take care of work-related matters outside of my working hours.	2
			In my free time, I am available to people I deal with professionally.	2
**Influence and Possibilities for Development**	Influence at Work	3	Do you have a large degree of influence on the decisions concerning your work?	1
			Can you influence the amount of work assigned to you?	1
			Do you have any influence on what you do at work?	1
	Degrees of Freedom (Breaks / Holidays)	2	Can you decide when to take a break?	1
			Can you take holidays more or less when you wish?	1
	Possibilities for Development	3	Is your work varied?	1
			Do you have the possibility of learning new things through your work?	2
			Can you use your skills or expertise in your work?	2
	Meaning of Work	2	Is your work meaningful?	2
			Do you feel that the work you do is important?	2
	Commitment to Workplace	2	Are you proud to be part of this organization?	2
			Do you enjoy telling others about your workplace?	2
**Social Relations and Leadership**	Predictability of Work	2	At your place of work, are you informed well in advance concerning for example important decisions, changes or plans for the future?	2
			Do you receive all the information you need in order to do your work well?	2
	Role Clarity	3	Does your work have clear objectives?	2
			Do you know exactly which areas are your responsibility?	2
			Do you know exactly what is expected of you at work?	2
	Role Conflicts	3	Are contradictory demands placed on you at work?	2

			Do you sometimes have to do things which ought to have been done in a different way?	2
			Do you sometimes have to do things which seem to be unnecessary?	2
	Quality of Leadership	3	Your immediate supervisor ensures good development opportunities for individual employees.	2
			Your immediate supervisor attaches great importance to job satisfaction.	2
			Your immediate supervisor plans the work well.	2
	Support at Work	4	How often do you get help and support from your colleagues, if needed?	1
			How often are your colleagues willing to listen to your problems at work, if needed?	1
			How often do you get help and support from your immediate superior, if needed?	1
			How often is your immediate superior willing to listen to your problems at work, if needed?	1
	Feedback	2	How often does your immediate superior talk with you about how well you carry out your work?	1
			How often do your colleagues talk with you about how well you carry out your work?	1
	Quantity of Social Relations	1	Can you chat with colleagues while you work?	1
	Sense of Community	2	Is there a good atmosphere between you and your colleagues?	1
			Is there good co-operation between the colleagues at work?	1
	Unfair Treatment	1	Do you often feel unfairly critizied, harassed, or embarrassed in front of others by colleagues and superiors?	1
	Trust and Justice	4	Does the management trust the employees to do their work well?	2
			Can the employees trust the information that comes from the management?	2
			Are conflicts resolved in a fair way?	2
			Is the work distributed fairly?	2
	Recognition	1	Is your work recognized and appreciated by the management?	2
**Additional Factors**	Work Environment / Phys. Demands	6	How often do you do heavy physical work, e.g., heavy lifting, carrying, or pushing?	1
			How often are you exposed to noise or loud ambient noise at your workplace?	1
			How often do you come into contact with chemicals or hazardous substances at work?	1
			How often are you exposed to drafts or extreme temperatures ar your workplace?	1
			How often are you exposed to poor air quality at work, e.g., cigarette smoke, gases, or similar?	1
			How often are you exposed to poor lighting conditions at work, e.g., bright or dim light?	1
	Job Insecurity	3	Are you worried about becoming unemployed?	2
			Are you worried about new technology making you redundant?	2

			Are you worried about it being difficult for you to find another job if you became unemployed?	2
	Insecurity over Working Conditions	3	Are you worried about being transferred to another job against your will?	2
			Are you worried about the timetable being changed (shift, weekdays, time to enter and leave…) against your will?	2
			Are you worried about a decrease in your salary?	2
**Effects**	Intention to leave Profession / Job	2	How often have you thought about quitting your job in the last 12 months?	3
			How often have you thought about changing jobs in the last 12 months?	3
	Job Satisfaction	7	Regarding your work in general. How pleased are you with:	
			- your work prospects?	4
			- the people you work with?	4
			- the physical working conditions?	4
			- the way your department is managed?	4
			- the way your abilities are used?	4
			- the challenges and skills involved in your work?	4
			- your job as a whole, everything taken into consideration?	4
	General Health	1	If you evaluate the best conceivable state of health at 10 points and the worst at 0 points: how many points do you then give your present state of health?	5
	Burnout Symptoms	3	How often have you been physically exhausted?	1
			How often have you been emotionally exhausted?	1
			How often have you felt worn out?	1
	Presenteeism	1	How often do you come to work even though you feel really sick and unwell?	1
	Inability to Relax	1	How often are you unable to forget about work during your free time?	1
	Work Engagement	3	How often do you experience the following?	
			At my work, I feel bursting with energy.	1
			I am enthusiastic about my job.	1
			I am immersed in my work.	1

***Response option:**
1: 1=always to 5=never
2: 1=to a very large exten to 5=to a very small extent
3: 1=never to 5 =every day
4: 1=very satisfied to 4=very unsatisfied
5: 0=worst possible state of health to 10=best possible state of health

Items with a 5-point Likert format (1=always … 5=never) are transformed to a 0 to 100 scale. The scale score is calculated as the mean of the items for each scale. If at least 50% of the items of a scale were answered, the scale value is calculated as the average of the items answered. If less than 50% of the items of a scale were answered, the scale value is regarded missing.
